# A Fluorescent Biosensor for Streptavidin Detection Based on Double-Hairpin DNA-Templated Copper Nanoparticles

**DOI:** 10.3390/bios13020168

**Published:** 2023-01-20

**Authors:** Qiangsheng Xiao, Mingjian Chen, Wanpin Nie, Fengjiao Xie, Xiao Yu, Changbei Ma

**Affiliations:** 1 Department of Hepatobiliary and Pancreatic Surgery, The Third Xiangya Hospital of Central South University, Changsha 410013, China; 2 School of Life Sciences, Central South University, Changsha 410017, China; 3 The Third Affiliated Hospital of Southern Medical University, Guangzhou 510640, China

**Keywords:** streptavidin, double-hairpin, copper nanoparticles, exonuclease

## Abstract

In this paper, we developed a sensitive, label-free and facile fluorescent strategy for detecting streptavidin (SA) based on double-hairpin DNA-templated copper nanoparticles (CuNPs) and terminal protection of small molecule-linked DNA. Herein, a special DNA hairpin probe was designed and synthesized, which contained two poly T single-stranded loops and a nick point in the middle of the stem. Inspired by the concept of the terminal protection interaction, the specific binding of SA to the biotinylated DNA probe can prevent the exonuclease degradation and keep the integrity of DNA probe, which can be used for synthesizing fluorescent CuNPs as a template. Conversely, the DNA probe would be digested by exonucleases and therefore, would fail to form CuNPs without SA. After systematic optimization, the detection range of SA concentration is from 0.5 to 150 nM with a low detection limit of 0.09 nM. Additionally, the proposed method was also successfully applied in the biological samples. Finally, the proposed method is sensitive, effective and simple, and can be potentially applied for predicting diseases and discovering new drugs.

## 1. Introduction

In recent years, the interactions between proteins and small molecules have been extensively studied for developing molecular diagnostics and discovering new drugs [1,2]. The detection method of DNA and proteins is based on the principle that the strong connections between proteins and small molecules at the terminal of single-stranded DNA (ssDNA) can prevent the degradation of ssDNA by exonuclease (Exo) [3,4]. This phenomenon, named terminal protection interaction, is convenient, inexpensive and easy to operate when used in biological samples [5,6]. The small molecule/protein interactions have been extensively exploited in protein assay in recent years [7,8].

Streptavidin (SA) obtained from Streptomyces avidinii has shown an extraordinarily high affinity for biotin [9]. SA is used in many aspects, such as biochemical sensors and nano biotechnology [8]. Its conjugation with enzyme or fluorescein is widely used in immunological detection assays. Due to the good biological resistance against extreme conditions, such as proteolytic enzymes, detergents (e.g., SDS), denaturants (e.g., guanidinium chloride) and extreme temperatures or pH, the SA-biotin complex is widely used in the fields of molecular biology [10]. The strong interactions between SA and biotin can be applied for biomedical diagnosis (predicting diseases and chemical genetics), target drug screening and molecular therapeutics. Therefore, it is crucial to develop a sensitive and facile strategy for SA detection [11,12].

Traditionally, different assays are available for the detection of SA, such as the protein-fragment complementation assay [13], near-field scanning optical microscopy [14], kinetic capillary electrophoresis [15], and surface plasmon resonance [16]. These traditional detection methods are usually highly sensitive and require a small amount of sample, but they also have some disadvantages, e.g., expensive instruments and a time-consuming and cumbersome assay, which limit their practical applications [17]. Focsan and Campu et al. reported an efficient and simple technique for detecting biotin-SA interactions with plasmonic nanobiosensors. Despite its high specificity, the limit of detection is still unsatisfactory in SA determination [18]. Khan et al. developed a highly sensitive SA detection method using liquid crystal, and the requirement for a special instrument is the major disadvantage for its application [19]. Therefore, a low-cost, highly sensitive and facile method needs to be developed for detecting SA.

DNA-templated fluorescent metal nanoparticles are new fluorescent probes with several advantages, such as facile synthetic process, good biological compatibility, outstanding optical properties and powerful signals [20,21,22]. Compared with other metal nanoparticles (i.e., silver nanoparticles and gold nanoparticles), copper nanoparticles (CuNPs) are extensively used in various bioanalysis due to their distinctive properties, such as faster synthesis, less toxicity, low-cost and lager MegaStokes shift [23,24,25]. As of now, CuNPs are used for developing various reporter systems for label-free sensing [26,27,28,29,30,31]. Wang et al. reported an efficient technique for microRNA detection using CuNPs based on rolling circle replication [32].

In this study, these distinct features of fluorescent CuNPs have been exploited for developing novel streptavidin detective sensors, based on double-hairpin DNA-templated CuNPs and terminal protection of small molecule-linked DNA. In this paper, a special DNA hairpin probe, which contained two poly T single-stranded loops and a nick point in the middle of the stem, was designed and synthesized for fluorescent CuNPs’ formation. In the absence of SA, Tn can be degraded by Exo I and III to become mononucleotide, which failed to act as a template in the synthesis of fluorescent CuNPs. Conversely, the strong interaction between streptavidin and biotin can protect the hairpin DNA even in the presence of Exo III and Exo I. Therefore, the reaction system would generate very high fluorescence intensity with the CuNPs, and the concentrations of SA can be associated with the fluorescence signal. Furthermore, the proposed method would be beneficial in the further research on developing protein detection methods, but also can be utilized as biosensors.

## 2. Materials and Methods

### 2.1. Materials and Reagents

Exonuclease I (Exo I), Exonuclease III (Exo III) were purchased from New England Biolabs Inc. (Ipswich, MA, USA). Streptavidin, human serum albumin (HSA), bovine serum albumin (BSA), lysozyme (Lyz), Immunoglobulin G (IgG) were bought from Sigma-Aldrich (St. Louis, MO, USA). The tris base, sodium chloride (NaCl), magnesium chloride (MgCl_2_), copper sulfate (CuSO_4_), sodium ascorbate (Vc) were bought from Sinopharm Chemical Reagent Co., Ltd. (Shanghai, China). The DNA probe was obtained from Shanghai Sangon Biotech Co. Ltd. (Shanghai, China). The sequence is as follows. The special DNA probe (Tn): 5′-TATATAGCTTTTTTTTTTTTTTTTTTTTTAGCTATATATATATA GCTTTTTTTTTTTTTTTTTTTTTAGCTATATA-biotin-3′.

### 2.2. Apparatus

The fluorescence intensity was determined by an F-2700 fluorescence spectrophotometer (Hitachi Ltd, Hitachi, Japan). The fluorescence emission spectra were recorded in the range of 530–650 nm with the excitation wavelength set at 340 nm. The excitation slit and emission slit were both set to 10 nm. The photomultiplier tube voltage was 700 V.

### 2.3. Optimization of the Experimental Conditions

For obtaining the best reaction systems, the reaction conditions, such as the concentrations of Tn, CuSO_4_, Vc and enzyme, and their reaction times were optimized. For optimizing Tn concentration, different concentrations of Tn were mixed with 500 nM SA in Tris-HCl buffer at 37 °C for 30 min. Then, 100 U/mL of Exo I and Exo III was fed into the reaction system and incubated for 30 min at 37 °C. Then, 100 μM of CuSO_4_ and 1 mM of Vc were added and incubated for 10 min at room temperature, utilizing the variation of fluorescence intensity to identify the optimal Tn concentration. The rest of the reaction conditions were optimized in a similar way.

### 2.4. Detection of SA

For quantifying the concentration of SA, different concentrations of SA were mixed with 200 nM of Tn in reaction buffer at 37 °C for 30 min, and then 30 U/mL of Exo I and 50 U/mL of Exo III were added and incubated for 30 min at 37 °C. Next, 80 μM of CuSO_4_ and 1 mM of Vc were added into the reaction system for 10 min at room temperature. An F-2700 fluorescence spectrophotometer was used to measure the fluorescence intensity. For verifying the application of this assay, different concentrations of SA in 1% human serum were measured.

### 2.5. Selectivity

SA was substituted by 150 nM of different proteins (i.e., BSA, HSA, Lyz, IgG and blank) for investigating the selectivity of the proposed method. The rest of the experimental steps were same in the above-mentioned method. At last, the fluorescence intensity can be measured at 600 nm with the excitation wavelength of 340 nm.

## 3. Results

### 3.1. Principle of the SA Detection

**Scheme 1:**
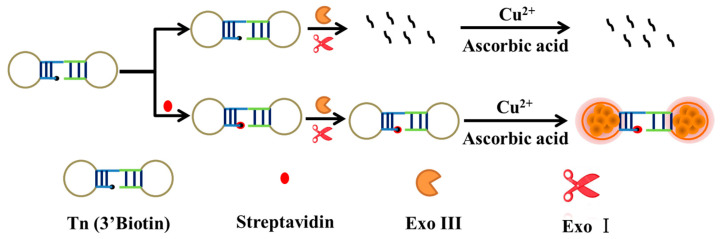
Schematic of streptavidin detection based on double-hairpin DNA-templated copper nanoparticles and terminal protection of small molecule-linked DNA.

The key part of the fluorescent biosensor is the formation of the SA-biotin complex, which can prevent degradation of the DNA probe (Tn) by the Exo I and Exo III with its strong steric hindrance [33]. At first, a special 3′-biotin-modified DNA probe was designed, which contained two poly T loops after forming the hairpin structure. In the absence of SA, Tn can be easily degraded into mononucleotide by exonucleases (Exo III for the double-stranded stem and Exo I for the remaining single-stranded loop) [34]. Therefore, no CuNPs were formed, and fluorescence did not generate. Conversely, in the presence of SA, due to the terminal protection of the SA-biotin complex, Tn retained the intact hairpin structure after adding Exo III and Exo I. Tn could be used as a template for CuNPs’ formation after adding CuSO4 and Vc, generating a high fluorescence intensity [35,36]. Therefore, the SA concentrations can be easily identified from the fluorescence change in the solutions (Scheme 1).

### 3.2. Feasibility Assessment of the SA Detection Assay

**Figure 1:**
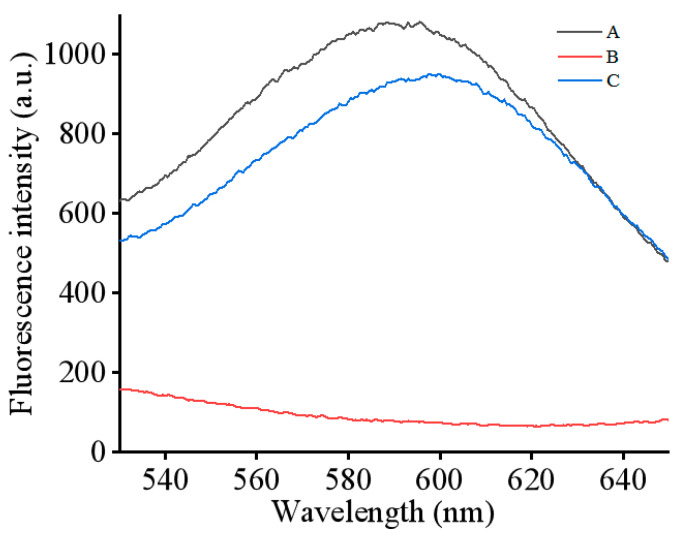
The feasibility of the proposed method. (A) Tn + CuSO_4_ + Vc; (B) Tn + Exo III + Exo I+ CuSO_4_ + Vc; (C) Tn + SA + Exo III + Exo I+ CuSO_4_ + Vc. Concentrations of Tn, SA, Exo III, Exo I, CuSO_4_ and Vc were 200 nM, 500 nM, 100 U/mL, 100 U/mL, 100 μM and 1 mM, respectively.

In order to evaluate the feasibility of the fluorescent biosensor, the fluorescence spectra were obtained under different conditions (Figure 1). A high fluorescence intensity was detected when the Tn probe was mixed with CuSO_4_ and Vc, indicating that CuNPs were synthesized successfully (Figure 1A). After the addition of Exo III and Exo I, the fluorescence intensity was diminished considerably due to the degradation of the enzyme (Figure 1B). As expected, when SA was added into the mixture before the enzyme, the fluorescence intensity increased dramatically (Figure 1C). The results suggested that the SA-biotin complex can prevent the catalysis of exonuclease [37]. Therefore, the proposed method can be used for the detection of SA.

### 3.3. Optimization of the Detection Strategy

The reaction conditions were optimized to obtain the best detection performance. (a) concentration of Tn; (b) concentration of Cu^2+^; (c) concentration of Vc; (d) concentration of Exo I; (e) concentration of Exo III; (f) reaction time of enzyme; (g) reaction time of SA. The best results of reaction conditions are as follows: (a) optimal Tn concentration: 200 nM (Figure S1 in Supplementary Materials); (b) optimal Cu^2+^ concentration: 80 μM (Figure S2); (c) optimal Vc concentration: 1 mM (Figure S3); (d) optimal concentration of Exo I: 30 U/mL (Figure S4); (e) optimal concentration of Exo III: 50 U/mL (Figure S5); (f) optimal reaction time of enzyme: 30 min (Figure S6); (g) optimal reaction time of SA: 30 min (Figure S7).

### 3.4. Performances of the Proposed Strategy

**Figure 2:**
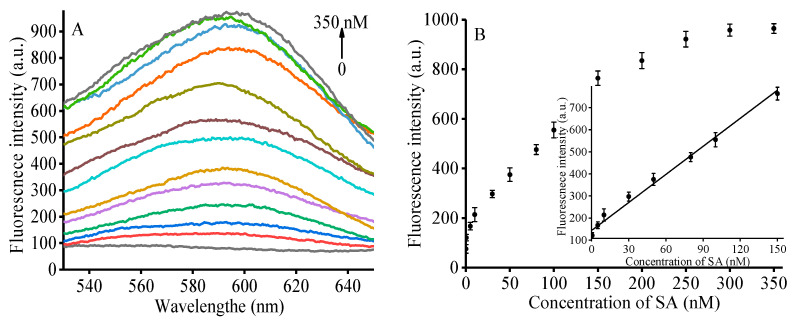
(**A**) Fluorescence emission spectra in the response to different concentrations of SA (0, 0.5, 5, 10, 30, 50, 80, 100, 150, 200, 250, 300, 350 nM) under optimal concentration. (**B**) Fluorescence intensity versus SA concentration at 600 nm and the linear relationship between fluorescence intensities and low concentrations of SA (inset); error bars were estimated from three replicate measurements.

Under the optimal reaction conditions, the fluorescence intensities at different concentrations of SA (0, 0.5, 5, 10, 30, 50, 80, 100, 150, 200, 250, 300, 350 nM) were recorded. The fluorescence intensity increased with the enhancement of SA concentrations (in Figure 2A). Figure 2B shows a good linear relationship in the concentrations from 0.5 to 150 nM. Moreover, the regression equation was Y = 4.0902X + 153.58 with an R^2^ of 0.9927 (where Y was the fluorescence intensity at 600 nm and X was the SA concentration, respectively). The LOD (detectable lowest concentration) was 0.09 nM (S/N = 3), which shows equivalent or better detection capacity than the previous works in the literature (Table 1). Compared with the previous work, this method is simple and rapid [32]. Therefore, this method has a wide detection range and a very low detection limit, which is a very promising SA detection method.

### 3.5. Selectivity of the Method

**Figure 3:**
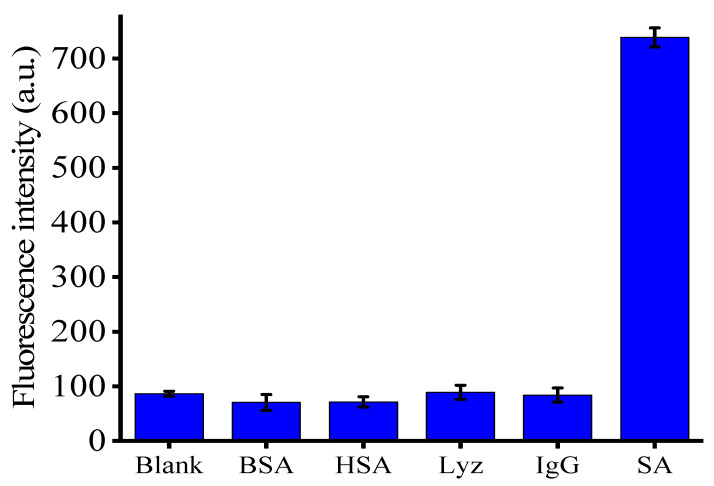
Fluorescence intensity of reaction system in the presence of SA, BSA, HSA, Lyz and IgG. Error bars were estimated from three replicated measurements.

We tested 150 nM of several proteins, such as BSA, HAS, IgG, Lyz and blank, by the proposed assay under the optimized concentrations to investigate the selectivity [38]. The fluorescence intensity of other protein samples, which is comparable to the blank one, is obviously different from the sample adding SA (in Figure 3). The reason is that there is a high specificity between SA and biotin, which can protect DNA from exonuclease degradation through the terminal protection interaction. Thus, only the group with SA has a high fluorescence signal. These results indicate that the proposed assay showed good selectivity for the quantitative determination of SA.

### 3.6. Practical Applicability of the Detection Strategy

In order to further verify the practical application value of this biosensor, we applied the fabricated sensing platform to the detection of SA in 1% human serum [39]. The human serum samples were provided by us. Three concentrations of SA (20, 60, 100 nM) were determined by the proposed assay in 1% human serum diluted by adding reaction buffer. We evaluated the recovery rates for different concentrations of SA (Table 2), such as 99% for 20 nM, 98.5% for 60 nM and 100.6% for 100 nM. Therefore, the proposed strategy can be successfully applied in biological systems for SA detection.

## 4. Conclusions

In summary, a simple and sensitive fluorescent method, based on the small-molecule-linked DNA terminal protection strategy and double-hairpin DNA-templated CuNPs, was successfully developed for SA determination. The fluorescent biosensor utilizes the specific binding between SA and biotin to prevent the degradation of enzymes and protect the formation of CuNPs, and then affect the change of fluorescence signal. This method exhibits a linear range from 0.5 to 150 nM with a low detection limit of 0.09 nM (S/N = 3). Overall, the detection assay has a wide linear range, low detection limit, and good specificity. In addition, the successful application of the method in the human serum demonstrates its practical use in biological systems. Thus, the proposed method is sensitive, label-free and facile, and can be potentially applied in biological systems.

## Figures and Tables

**Table 1:** Comparison of different methods for the determination of SA.

Method	Material	Detection Range (nM)	LOD (nM)	Reference
SPR	Gold nanoparticle		5	18
Electrochemistry	Gold electrode	0.5–5000	0.02	8
Fluorescence	SYBR Green I	0.01–0.1	0.02	1
Fluorescence	AgNCs	6–600	2.6	9
Fluorescence	SYBR Green I	5–200	0.4	10
Fluorescence	ThT	0.1–17	0.07	11
Fluorescence	CuNCs	1–200	0.47	12
Fluorescence	Pyrene	4–1000	1.07	17
Fluorescence	FAM	0.15–12	0.08	33
Fluorescence	CuNPs	0.5–150	0.09	This work

**Table 2:** Recovery experiments of SA in diluted human serum using this method (*n* = 3).

Sample	Added (nM)	Found (nM)	Recovery (%)	RSD (%)
1	20	19.8 ± 0.4	99	7.71
2	60	59.1 ± 1.5	98.5	9.31
3	100	100.6 ± 2.1	100.6	6.05

## Data Availability

Not applicable.

## Supplementary Materials

The following supporting information can be downloaded at: https://www.mdpi.com/article/10.3390/bios13020168/s1, Figure S1: Optimization of the concentration of Tn; Figure S2: Optimization of the concentration of Cu^2+^; Figure S3: Optimization of the concentration of Vc; Figure S4: Optimization of the concentration of Exo I; Figure S5: Optimization of the concentration of Exo III; Figure S6: Optimization of the reaction time of enzyme; Figure S7: Optimization of the reaction time of SA.
