# Supplementary material for: A Fluorescent Biosensor for Streptavidin Detection Based on Double-Hairpin DNA-Templated Copper Nanoparticles
Source: Biosensors (Basel). 2023 Jan 20;13(2):168. doi: 10.3390/bios13020168 (PMC9953726; doi:10.3390/bios13020168)
Supplement: Supplementary file 1 [file biosensors-13-00168-s001.zip › biosensors-2110905-supplementary.pdf]

Supporting Material

A Fluorescent Biosensor for Protein Detection Based on Double-Hairpin DNA-Templated Copper Nanoparticles

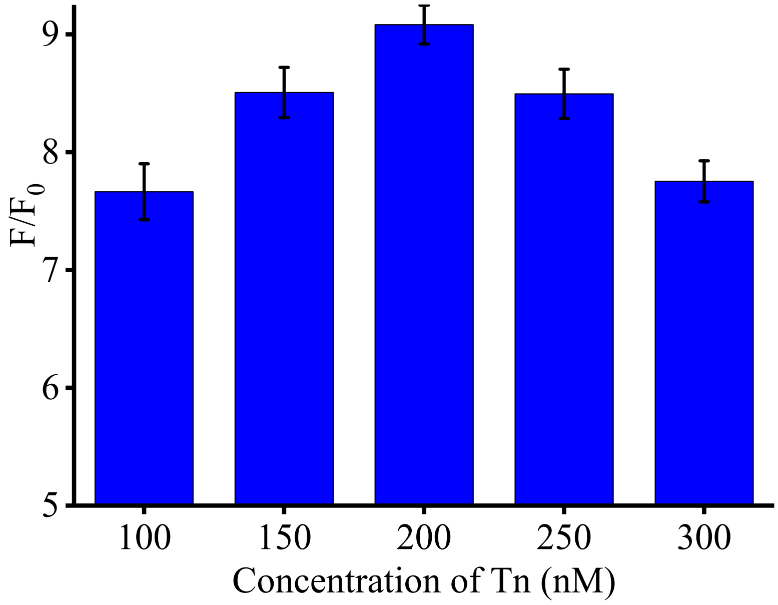

Figure S1 Optimization of the concentration of Tn (100, 150, 200, 250, 300 nM). The increments of fluorescence intensity measured at different concentrations of the Tn were expressed as F/F_0 ratios, where F and F_0 represent the fluorescence intensities detected in the presence and absence of SA, respectively. Error bars were estimated from three replicate measurements.

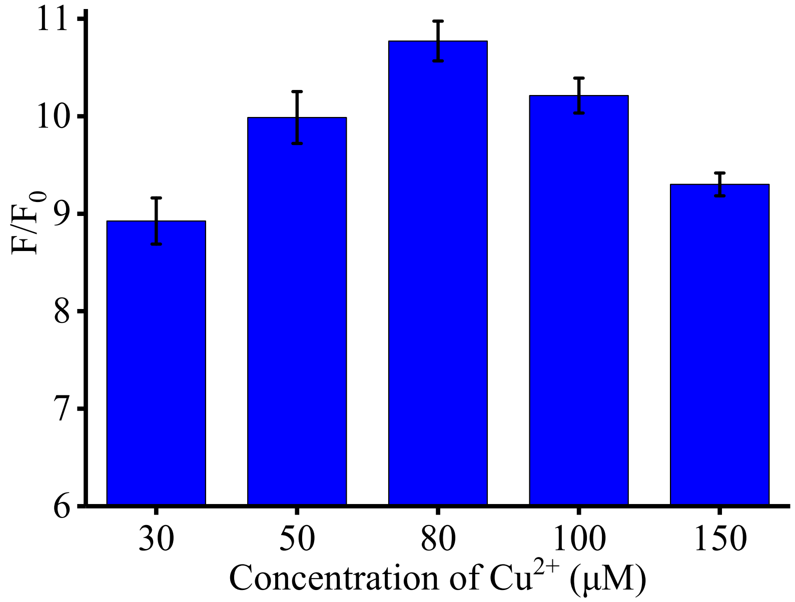

Figure S2 Optimization of the concentration of Cu^{2+} (30, 50, 80, 100, 150 μM). The increments of fluorescence intensity measured at different concentrations of Cu^{2+} were expressed as F/F_0 ratios, where F and F_0 represent the fluorescence intensities detected in the presence and absence of SA, respectively. Error bars were estimated from three replicate measurements.

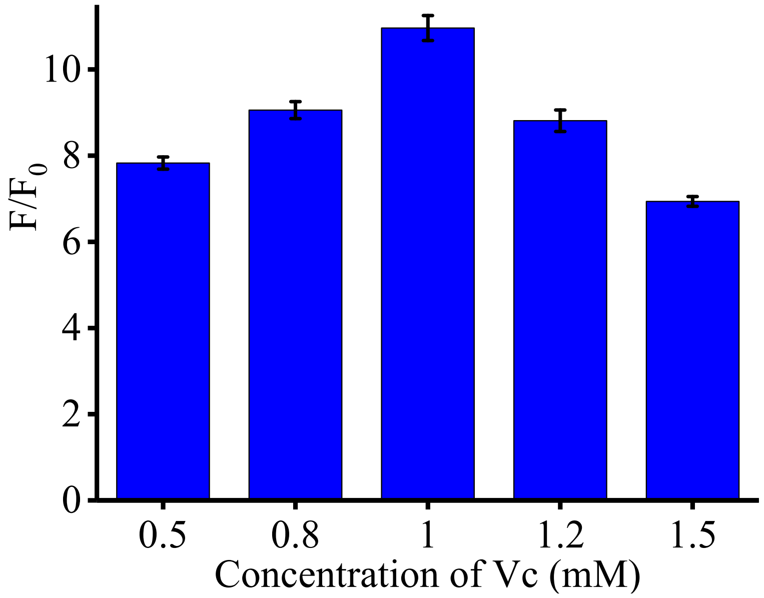

Figure S3 Optimization of the concentration of Vc (0.5, 0.8, 1, 1.2, 1.5 mM). The increments of fluorescence intensity measured at different concentrations of Vc were expressed as F/F_0 ratios, where F and F_0 represent the fluorescence intensities detected in the presence and absence of SA, respectively. Error bars were estimated from three replicate measurements.

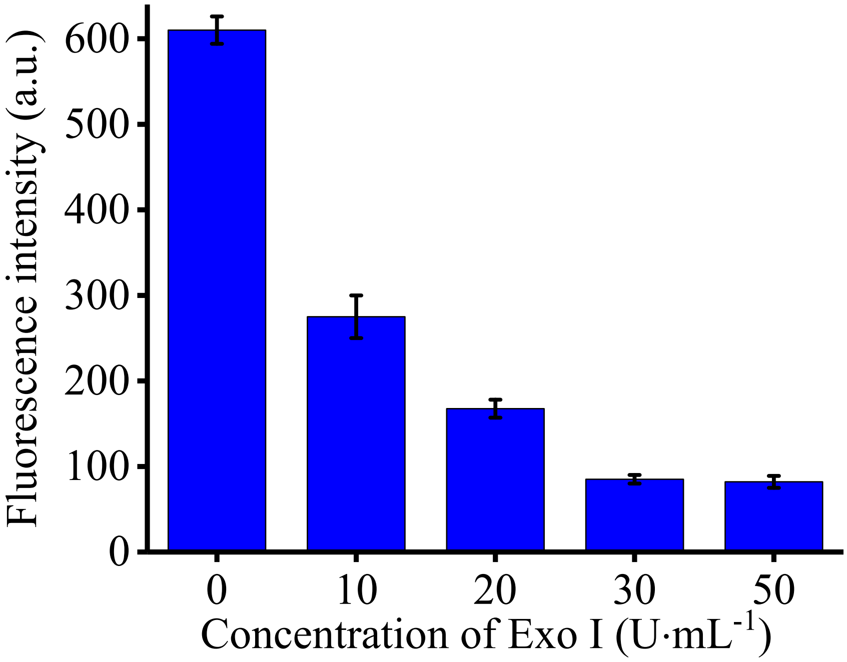

Figure S4 Optimization of the concentration of Exo I (0, 10, 20, 30, 50 U·mL^{-1}). Error bars were estimated from three replicate measurements.

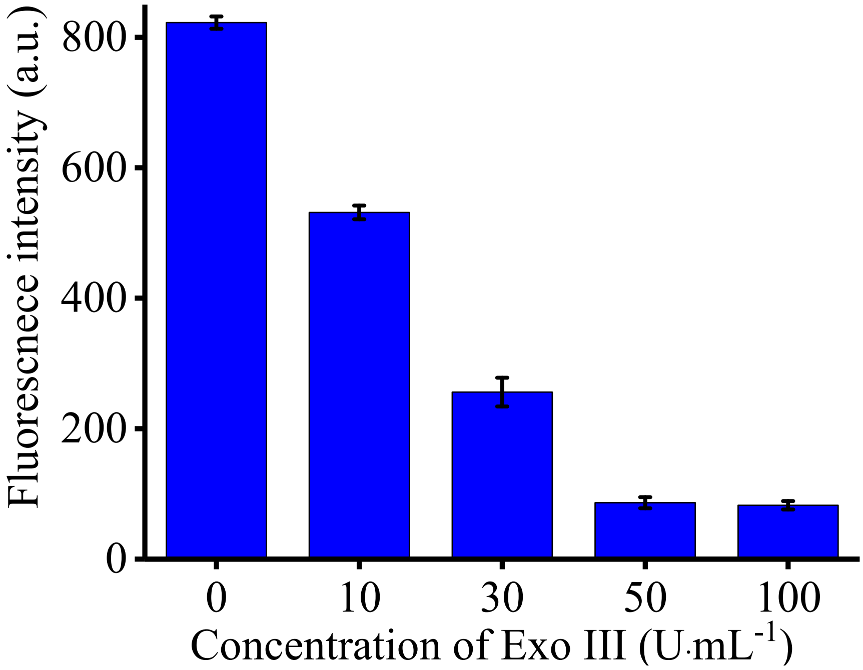

Figure S5 Optimization of the concentration of Exo III (0, 10, 30, 50, 100 U·mL^{-1}). Error bars were estimated from three replicate measurements.

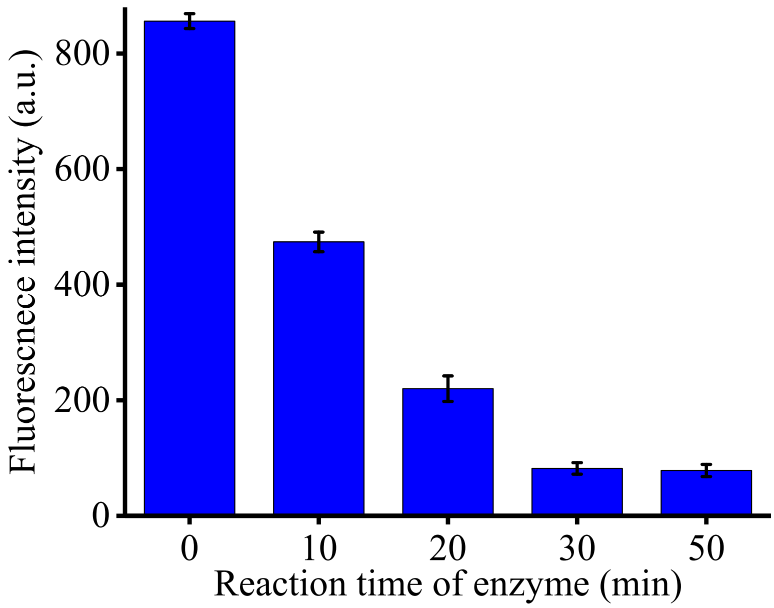

Figure S6 Optimization of the reaction time of enzyme (0, 10, 20, 30, 50 min). Error bars were estimated from three replicate measurements.

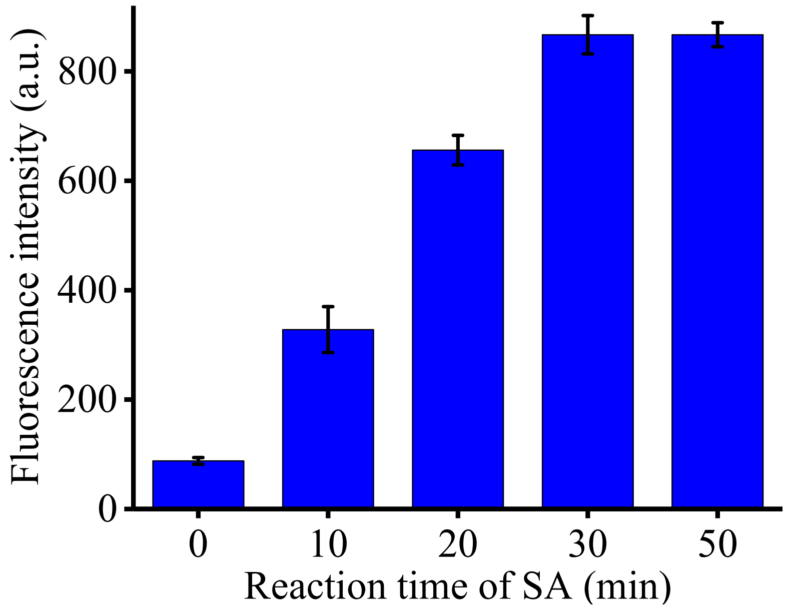

Figure S7 Optimization of the reaction time of SA (0, 10, 20, 30, 50 min). Error bars were estimated from three replicate measurements.
